# The trigger for pancreatic disease: NLRP3 inflammasome

**DOI:** 10.1038/s41420-023-01550-7

**Published:** 2023-07-14

**Authors:** Tianming Liu, Qiang Wang, Zhiwei Du, Lu Yin, Jiachen Li, Xianzhi Meng, Dongbo Xue

**Affiliations:** 1 Department of General Surgery, The First Affiliated Hospital of Harbin Medical University, Harbin, 150001 China; 2 Key Laboratory of Hepatosplenic Surgery, Ministry of Education, The First Affiliated Hospital of Harbin Medical University, Harbin, 150001 China

**Keywords:** Inflammasome, Mechanisms of disease

## Abstract

NLRP3 inflammasome is a multiprotein complex expressed in a variety of cells to stimulate the production of inflammatory factors. Activation of NLRP3 inflammasome depends on a complex regulatory mechanism, and its pro-inflammatory function plays an important role in pancreatic diseases. In this literature review, we summarize the activation mechanism of NLRP3 and analyze its role in each of the four typical pancreatic diseases. Through this article, we provide a relatively comprehensive summary to the researchers in this field, and provide some targeted therapy routes.

## Facts

- Activation of NLRP3 inflammasome is thought to occur by three different pathways: the canonical pathway, the non-canonical and the alternative pathway.
- NLRP3 inflammasome converts pro-IL-1β and pro-IL-18 into biologically active IL-1β and IL-18 by activating caspase-1 activity, which plays a pro-inflammatory role.
- The pro-inflammatory mechanism of NLRP3 inflammatories is related to pyroptosis.
- In the course of diabetes, NLRP3 inflammasome plays a pro-inflammatory role by disrupting β cell function.
- The search for effective new drugs to inhibit the action of NLRP3 inflammasome is a promising direction for the future treatment of pancreatic diseases.

## Open questions

- What signals regulate the priming and activation of NLRP3 inflammasome?
- What role does NLRP3 inflammasome play in acute and chronic pancreatitis?
- Whether NLRP3 inflammasome plays a different role in pancreatic cancer than in inflammation?

## Introduction

Since the 21st century, people have undoubtedly made significant progress in the field of pancreatic disease in pathophysiology, diagnosis, and treatment. However, the mortality rate of pancreatic disease has barely decreased over the past few decades, and its expensive consumption is also unacceptable [1]. With the development of molecular biology, more and more molecular mechanisms related to the pathogenesis of pancreatic disease have been studied.

The inflammasome is multiprotein complexes assembled by intracytoplasmic pattern recognition receptors (PRRs). After these pattern recognition receptors recognizing corresponding ligand, the downstream signaling pathway will be activated and promote the upregulation of inflammatory cytokines and chemokines, thereby regulating the inflammatory response. Among them, the leucine-rich repeat NOD-like receptors (NLRs), mainly expressed by myeloid cells, are the most common PRRs. Activation of the NLRP3 inflammasome in the NLRs family can trigger an inflammatory response.

The NLRP3 inflammasome has been found to express in various cells such as monocytes, neutrophils, dendritic cells, lymphocytes, osteoblasts, and epithelial cells. In immune cells, strong expression of NLRP3 was detected in neutrophils, monocytes, and dendritic cell, which are thought to be high producers of IL-1β. Interestingly, the expression of NLRP3 in neutrophils requires stimulation. NLRP3 was well expressed in B lymphocytes and T lymphocytes. The NLRP3 inflammasome plays a key role in the first line of defense against invading pathogens and possible allergens.

In recent years, researchers have found that there is a particular relationship between the occurrence and development of pancreatic diseases and inflammasomes [2]. Although there are more than one inflammasome associated with pancreatic diseases, such as absent in melanoma 2 (AIM2) inflammasome, which can also promote the release of IL-1β and IL-18 and lead to pancreatic inflammatory response. NLRP3, as a more widely studied inflammasome, is the main driving force of inflammation. Meanwhile, no article summarizes the relationship between pancreatic diseases and NLRP3 inflammasomes. We will review the effects of NLRP3 inflammasome on pancreas-related diseases (acute pancreatitis, diabetes, chronic pancreatitis, and pancreatic cancer), in order to provide insights into the pathogenesis of pancreatic diseases for researchers.

## The structure and effect mechanism of NLRP3 inflammasome

### Structure of NLRP3 inflammasome

NLRs generally consist of three parts: an N-terminal effector domain, a central nucleotide-binding domain (NBD/NOD/NACHT), and C-terminal leucine-rich repeats (LRR). Among NLRs, NACHT and LRR domains are the common features of all NLR proteins except NLRP10, but the N-terminal effector domains are variable. Therefore, the type and outcome of the regulation of NLRs activation can be influenced by the N-terminal effector domain. In all NLRs, N-terminal effector domains include pyrin domain (PYD) domains, caspase activation and recruitment domain (CARD) domains, and baculovirus inhibitory repeat domains. We can further subdivide NLR proteins based on various N-terminal effector domains.

The NLRP3 inflammasome is thought to be composed of an upstream receptor NLR family protein, an adapter protein named ASC (apoptosis-associated speck-like protein containing a CARD domain) acting as a bridge, and a downstream effector protein pro-caspase-1. The N-terminal effector domain of NLRP3 is the PYD domain, which is a typical PYD receptor protein. The N-terminus of the ASC interact with that of the NLR through the PYD domain to bind with each other and form a homotypic PYD: PYD. Furthermore, the C-terminal CARD is released from its self-inhibited conformation to recruit pro-caspase-1 through the homotypic CARD-CARD interaction, and aggregation of pro-caspase-1 results in autoproteolysis and generation of enzymatically active caspase-1 [3]. When pathogen-associated molecular patterns (PAMPs) or damage-associated molecular patterns (DAMPs) stimulate the receptor protein, it assembles and exerts its effect through the LRR-NACHT-PYD: PYD-CARD-CARD-pro-caspase 1 axis. High concentrations of pro-caspase 1 facilitates its heterodimerization, self-cleavage, and activation, thereby activating caspase-1 [4–6]. Active caspase-1 converts the cytokines pro-IL-1β and pro-IL-18 into the mature bioactive forms IL-1β and IL-18 [7].

### The mechanism of action of NLRP3 inflammasome

#### The canonical pathway

**Fig. 1:**
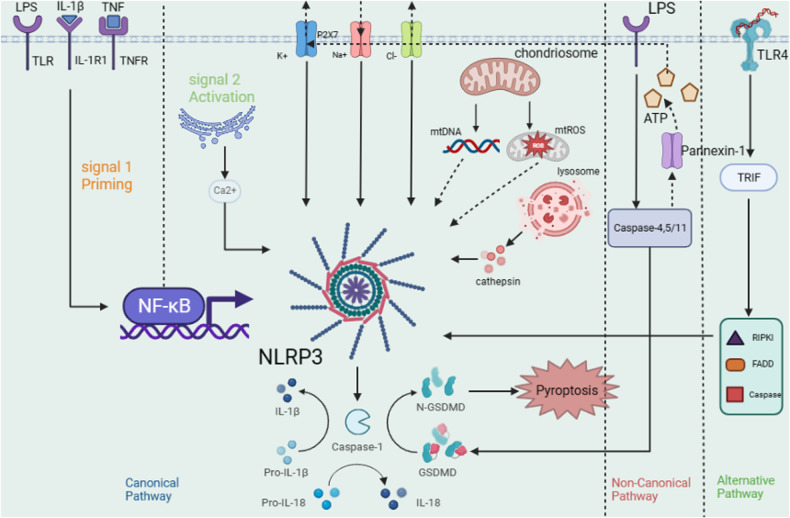
Activation pathway of NLRP3 inflammasome: generally, it can be divided into the canonical pathway; the non-canonical pathways and the alternative pathway. In canonical pathway,there are two steps which can activate NLRP3 inflammasome. The ultimate goal of these three pathways is to promote the secretion of IL-18 and IL-1β from NLRP3 inflammasome.

As Fig. 1 shows, the canonical pathway is generally divided into priming (signal 1) and activation (signal 2). The amount of NLRP3 in cells in a quiescent state is insufficient to activate the production of inflammasomes directly, and the levels of ASC and caspase-1 are stable. The canonical pathway holds that inflammasome cannot be produced by stimulating the body activation signal without the priming signal [8]. Typical priming signals (signal 1) receptors are interleukin 1 receptor type I (IL-1R1), Toll-like receptors (TLRs), and tumor necrosis factor receptor (TNFR). These receptor signals can lead to activation of the transcription factor NF-κB and upregulation of NF-κB-dependent expression levels of NLRP3 and pro-IL-1β [9].

NLRP3 can be indirectly activated by many pathogenic and sterile inflammatory signals. These signals include bacterial and viral PAMPs such as LPS and pathogen RNA [10]; pore-forming bacterial toxins such as melanin and gramin; extracellular adenosine triphosphate (ATP), reactive oxygen species, heme [11], and various metabolic crystals [12]. These agonists trigger specific activation of NLRP3, assembly of the inflammasome complex, and ultimately caspase-1 activation. Three significant signals that have been proven to activate the NLRP3 inflammasome include four ion fluxes, mitochondrial dysfunction and reactive oxygen species (ROS) production, and lysosomal damage.

K^+^ efflux is the earliest known and widely recognized NLRP3 activator [13]. Some familiar NLPR3 activating substances such as melanin, gramin, valinomycin, ATP, granule molecules, and cry can promote K^+^ efflux [14]. However, substances such as CL097 and imiquimod can promote the activation of NLRP3 inflammasomes by inducing the production of ROS rather than the potassium efflux mechanism [15]. This shows that K^+^ efflux is not necessary for NLRP3 activation but is still important. Increased cytosolic Ca^2+^ is especially significant for NLRP3 inflammasome activation, as inhibition of endoplasmic reticulum (ER) or plasma membrane Ca^2+^ channels attenuates caspase-1 activation and IL-1β secretion in response to NLRP3 stimulation. At the same time, the increase of Ca^2+^ can promote the interaction between NLRP3 and ASC and may affect the mitochondrial Ca^2+^ load, leading to mitochondrial dysfunction, generating mitochondrial ROS (mtROS), and thus affecting the activation of NLRP3 [16]. We believe that these two channels are important, but neither of them is indispensable in the activation of the NLRP3 inflammasome.

Na^+^ influx and Cl^-^ efflux: Since Na^+^ influx alone cannot activate the NLRP3 inflammasome, therefore, the relevant literature is less descriptive. However, it has been shown that increased Na^+^ influx could regulate NLRP3 inflammasome activation by reducing the threshold of K^+^ efflux [14]. Chloride channels, including the volume-regulated anion channel (VRAC) and the chloride intracellular channel (CLIC), are thought to regulate NLRP3 inflammasome activation by promoting NLRP3-Nek7 interaction [17]. Among these, CLIC-dependent chloride ion efflux is a downstream event of the potassium efflux-mitochondrial ROS axis, and CLIC-mediated chloride efflux can promote NEK7–NLRP3 interaction and subsequent ASC oligomerization. Nek7 is thought to be involved in forming or providing a common signal that acts upstream of NLRP3 and transmits that signal to its adaptation protein ASC. It may also mediate NLRP3 polymerization and point-like ASC aggregation [18]. On the other hand, through its function as a microtubule dynamics regulator, Nek7 may also facilitate the interaction of NLRP3 and ASC [19]. At the same time, decreased concentration of extracellular Cl^-^ can enhance ATP-induced caspase-1 activation and IL-1β maturation and secretion [20].

Mitochondria are generally thought to influence inflammasome activation either through ROS generation or through interaction with NLRP3 inflammasome components [21]. Although mtROS and oxidized mtDNA (mitochondrial DNA) produced by dysfunctional mitochondria are widely believed to be essential activators of NLRP3 inflammasome activation. Iyer and associates found ROS production induces NLRP3 inflammasome activation in a manner dependent on mitochondrial dysfunction [22], suggesting that ROS production is not a requirement for inflammasome activation.

Some particulate or crystalline substances, such as silica, calcium crystals, and cholesterol crystals, are engulfed by cells through the destruction of lysosomes, resulting in the release of lysosomal contents, such as lysosomal cathepsin B, together with lysosomal calcium signaling to regulates the release of IL-1β, which induces the activation of NLRP3 inflammasome [23]. At the same time, the lysosomal damage-mediated release of cathepsins and other factors can also activate the NLRP3 inflammasome by acting on the cell membrane to cause K^+^ efflux.

#### Influence of pyroptosis on canonical pathways

Pyroptosis is characterized by dependence on inflammatory caspase (primarily caspase-1, 4, 5, 11), accompanied by the release of a large number of pro-inflammatory factors. Involves the formation of cell membrane pores mediated by gasdermin proteins, causing ion transport, accompanied by inflammation and immune responses. The ion imbalance causes cells to swell, dissolve, and release pro-inflammatory factors, including interleukin-1β (IL-1β), interleukin-18 (IL-18), ATP, and the high mobility group box 1 (HMGB1) protein.

NLRP3 inflammasome mediates pyroptosis through cleaving gasdermin (GSDM) proteins, while GSDMD is the main substrate of NLRP3 inflammasome-induced pyroptosis. GSDMD has an N-terminal prereforming domain and a C-terminal self-suppression domain [24]. After PAMPs and DAMPs trigger NLRP3, NLRP3 promotes self-cleavage of pro-caspase-1 to produce activated caspase-1, thereby transforming pro-IL-1β and pro-IL-18 into the mature bioactive forms of IL-1β and IL-18 [25]. The activated caspase‐1 splits the GSDMD protein molecule and initiates oligomerization of the 31 kDa amino terminal portion (GSDMD-N) to form the membrane pore. This promotes the secretion of inflammatory factors and cell infiltration.

#### Non-canonical and alternative pathways

Non-canonical activation of the NLRP3 inflammasome is primarily initiated by the LPS of Gram-negative bacteria, which can be recognized by caspase-11 in mice (or caspase-4/5 in humans) through direct interaction, leading to caspase -11 autoproteolysis and activation. Activated caspase-11 also cleaves GSDMD to induce membrane pore formation and pyroptosis. The GSDMD protein is cut into two independent domain fragments at the N-terminus and C-terminus [26]. This leads to changes in cell osmotic pressure that lead to cell membrane rupture and cell death. Unlike canonical pathways, caspase-1 can process pro-IL-1β and pro-IL-18, but caspase-11 cannot [27]. In addition to GSDMD, it can also activate pannexin-1 through caspase-11 to release ATP to activate the P2X7 receptor (P2X7R) [28]. P2X7R, acting as an ATP-gated cation-selective channel, opens a pore that triggers K+ efflux and Induces K+ efflux, which drives NLRP3 inflammasome assembly and IL-1β release.

The alternative pathway is a newly proposed concept of inflammasome activation, which is considered having no concern with K^+^ efflux and induction of apoptosis. A recent study suggested that this NLRP3 activation is induced through the RIPK1, FADD, and caspase-8 pathways downstream of TLR4-TRIF signaling [29]. Similarly, Gaidt also proposed that TLR4-TRIF-RIPK1 - FADD-CASP8 signaling is involved in this alternative pathway to promote NLRP3 inflammasome activation, so they speculate that caspase-8-mediated activation of an unknown intermediate protein is indispensable for alternative inflammasome activation [30].

## The role of NLRP3 inflammasome in acute pancreatitis

### The pro-inflammatory mechanism of NLRP3 inflammasome in acute pancreatitis

The mechanism of acute pancreatitis (AP) is the self-digestion of pancreatic tissue caused by the increase in the level of pancreatic enzymes or premature activation caused by various reasons, which leads to pancreatic acinar cells damaged. IL-1β and IL-18 are typical cytokines that promote pancreatic inflammation, but because they are synthesized as precursor proteins, they depend on the proteolytic activity of caspase-1 to produce their bioactive forms. Here again, NLRP3 is required to convert caspase-1 into an activated state. The mechanism of NLRP3 inflammasome in pancreatitis can be regulated by the central transcription factor NF-κB on the expression of known pancreatitis-related genes such as pro-inflammatory factors IL1β, IL6, IL8, IL18, tumor necrosis factor α (TNFα) and chemokine MCP-1 [31].

IL1β, produced in response to activation of the NLRP3 inflammasome, can be transcribed by monocytes, macrophages, and dendritic cells, and is considered the main cytokine that mediates early inflammation and proliferation to extrapancreatic tissues during AP. IL-1β induces autophagy and reduces cell viability of pancreatic acinar cells by affecting calcium homeostasis and inducing trypsinogen activation in pancreatic acinar cells [32]. Similarly, IL18, expressed by macrophages, epithelial cells, and dendritic cells and stored in the cytoplasm, also plays a vital role in acute pancreatitis. In addition to being cleaved by caspase-1 to exert anti-inflammatory effects, IL18 can also function by linking IL18 receptor (IL18R) α and β, recruiting MyD88 and leading to the activation of NF-κB and mitogen-activated protein kinase (MAPK) [33]. Dual roles of IL-18 in T cell differentiation, IL-18 together with IL-12 enhances Th1 differentiation, but in the absence of IL-12, it induces Th2 cell responses. IL-18 can induce neutrophil migration and maturation during pancreatitis and plays a crucial role in activating endothelial cells [34].

Different from IL-1β and IL-18, although IL-33 is also involved in the pathway of inflammasome-induced pancreatitis, it does not require the activation of the inflammasome because caspase-1 will deactivate IL-33 [35]. Interestingly, similar to IL-18, IL-33 signaling activates the MyD88/Interleukin-1 receptor-associated kinases (IRAK)/TNF receptor-associated factor 6 (TRAF6) axis, activating downstream NF-κB and MAPK signaling pathways to promote NLRP3 production [36]. Nevertheless, some data show that IL-33 has a protective effect in AP [37, 38]. Therefore, its anti-inflammatory effect is not yet apparent, and further experiments are needed to confirm it.

### Factors affecting the effect of NLRP3 in acute pancreatitis

#### Related DAMP signals

Associated DAMP signals, which includes HMGB1, heatshockprotein70 (HSP70), ATP, and mtDNA, induce acute pancreatitis by affecting the NLRP3 inflammasome. HMGB1 is a widely distributed and highly conserved nuclear protein that may function as a DAMP to exacerbate pancreatitis damage through TLR4 and TLR9. Among them, TLR4 leads to the release of mtDNA during pancreatic injury, thereby accelerating the activation of NLRP3 inflammasome [39], which in turn induces acute pancreatitis. In recent years, studies have also found that HMGB1 can contribute to pancreatic damage by activating the neutrophil extracellular trap and then inducing the processing and expression of IL-1β in the pancreas [40].

As a typical protein, HSP70 also affects the inflammasome pathway. Asea found that extracellular HSP70 interacts with TLR and CD14 and regulates the immune response through the MyD88/IRAK/NF-κB signal transduction pathway which is similar to pathway of NLRP3-induced pancreatitis [41]. Song has found that recombinant HSP70 can aggravate AP in a TLR4-dependent manner in a mouse model [42].

Extracellular ATP released from damaged cells interacts with P2X7 to induce mitochondrial dysfunction, and P2X7 acts as an activator of the NLRP3 inflammasome, thus leading to inflammasome production, caspase-1 activation and secretion of IL1β and IL18 [3]. Intracellular and extracellular mtDNA play different roles. Intracellular mtDNA is easily oxidized and transferred to the cytoplasm, where it directly binds to NLRP3 to activate the NLRP3 inflammasome; extracellular mtDNA acts as a DAMP to participate in the initiation and activation of the NLRP3 inflammasome [43].

#### Cathepsins

Cathepsins are thought to induce acute pancreatitis by affecting the NLRP3 inflammasome. Lysosome-released cathepsin B (CTSB) is a cysteine protease localized in lysosomes [44]. Increaseing expression of activated CTSB aggravates injury and inflammation of pancreatic tissue. During the development of AP, CTSB not only activates the NLRP3 inflammasome pathway, which in turn induces caspase-1 activation and subsequent IL-1β, and IL-18 secretion, leading to inflammation, but also induces acinar cell death through pyroptosis, aggravating pancreatitis [45]. Furthermore, the release of cathepsins and other factors mediated by lysosomal damage can also activate the NLRP3 inflammasome by acting on the cell membrane to cause potassium efflux.

### Inflammasome pathway inhibitors in experimental acute pancreatitis

Since inflammasomes plays an essential role in the occurrence and development of acute pancreatitis, inhibiting the formation of inflammasomes and their pathways and pro-inflammatory effector molecules has become a hotspot in the treatment of experimental acute pancreatitis (Table 1).

**Table 1:** NLRP3 inflammasome pathway therapy for experimental acute pancreatitis.

DRUG	TYPE	THERAPY PATHWAY	PMID
Annakinra	Recombinant IL1 receptor antagonist anakinra	Inhibit IL-1	24912987
Rilonacept	Soluble IL-1β receptor	Weaken the function of IL-1β	20074281
Canakinumab	IL-1β monocolonal antibody	Weaken the function of IL-1β	26284424
MCC950	Sulfonylureas	Eliminate the oligomerization of ASC	31593700
Emodin	Natural plant extract	Inhibit P2X7/NLRP3 pathway	20572302
Danshensu	Natural plant extract	Inhibit NLRP3 inflammasome, NF-κB pathway, and STAT3 pathway	30425719
Fraxinellone	Natural plant extract	Inhibit caspase-1, IL-1β and NLRP3	30716587
Withferin A	Natural plant extract	Inhibit NF-κB and the activation of NLRP3 inflammasome	27418337
Rutin	Natural plant extract	Decrease the expression levels of ASC, TNF-α, and IL-1β, and inhibit the activation of caspase-1	25060908
Cordycepin	Natural plant extract	Inhibit the activation of NF-κB and NLRP3 inflammasome	32268154
Apocynin	Natural plant extract	Inhibit the activation of NLRP3, pro-caspase-1, and IL-1β	31437787
Indomethacin	Inhibitor of COX-2	Inhibit the activation of NLRP3 inflammasome	28867183
Iguratimod	Inhibitor of COX-2	Inhibit NF-κB pathway and activation of NLRP3 inflammasome	31541854
INT-777	Agonist of bile acid receptor	Block out ROS/NLRP3 pathway	29859191
Butyrate	Butyrate	Inhibit the activation of NLRP3 inflammasome	31347703
β-hydroxubutyric	Water-soluble ketone body	Decrease the expression levels of caspase-1, IL-1β, and NLRP3	34528884
Vancomycin, neomycin, and polymyxin-b	Antibiotic	Inhibiting the pancreatic NLRP3 pathway	31615312
3,4-DAA	O-aminobenzoic acid derivatives	Inactivating NF-κB and NLRP3 inflammasome signaling pathway	33319359

#### IL1 antagonists

Three typical IL-1 antagonists have been used clinically, namely: the recombinant IL-1 receptor antagonist Anakinra, the soluble decoy IL-1β receptor Rilonacept, and the neutralizing IL-1β antibody Canakinumab. These drugs can inhibit inflammation by inhibiting inflammasome generate an active IL family. The most typical drug is Annakinra, which can significantly reduce coronary protein-related pancreatic tissue damage and pancreatic apoptosis in rats [46]. This was the first IL-1 inhibitor designed and was approved by the Food and Drug Administration (FDA) for the treatment of patients in 2001 [47], but it has not been documented for the treatment of acute pancreatitis. The use of such drugs to prevent pancreatitis is still in the animal testing stage.

#### MCC950

MCC950, as the most effective selective inhibitor of NLRP3 inflammasome [48], reduces the formation of NLRP3 inflammasome mainly by eliminating ASC oligomerization. MCC950 has the ability to block NLRP3 inflammasome activation and IL-1β production by abolishing ASC oligomerization. And has also been proven to not only block local and systemic immune responses but also alleviate the severity of the disease [34].

#### Natural plant extracts

Some natural plant extracts have also been applied to block NLRP3 inflammasomes, among which the typical one is Emodin, which can delay the progression of AP by inhibiting P2X7/NLRP3 signaling pathway, thereby ameliorating the associated systemic inflammation [49]. As a flavonoid with anti-inflammatory, anti-allergic, anti-viral, and antioxidant properties, Rutin inhibits the autoactivation of trypsin and inhibits caspase-1 by attenuating the expression of ASC, TNF-α and IL-1β activation of NLRP3 inflammasomes, which can reduce pancreatic inflammation [50]. Danshensu, a Chinese herb, can inhibit the inflammatory response of AP by inhibiting the activation of NF-κB, STAT3, and NLRP3 inflammasome [51].

#### Cyclooxygenase-2 inhibitors

In recent years, it has been found that cyclooxygenase-2 (COX-2) inhibitor is one of the effective regulators of inflammatory response and NLRP3 inflammasome activation [52]. Indomethacin, the most common non-steroidal anti-inflammatory drug (NSAIDs) and cyclooxygenase-2 (Cox-2) inhibitor, can inhibit the activation of NLRP3 inflammasome and inflammatory response, and reduce the expression of IL-1β to protect the pancreas from damage [53]. Another COX-2 inhibitor, Iguratimod (T-614), was confirmed to inhibit the NF-κB signaling pathway and NLRP3 inflammasome activity [54].

#### Other substances that affect the formation of NLRP3 inflammasome

Some other substances can also affect the formation of NLRP3 inflammasome in pancreatitis. For example, the bile acid receptor agonist INT-777 effectively relieves inflammation and pancreatic acinar cell damage by blocking the ROS/NLRP3 pathway; ABX combination therapy (vancomycin, neomycin, and polymyxin b) attenuated the activation of TLR4 and NLRP3 by inhibiting the translocation of gut bacteria to the pancreas, thereby suppressing uncontrolled diffuse inflammation in the pancreas and adjacent organs during AP; β-hydroxybutyric acid can reduce the activity of caspase-1 and inhibit the maturation of IL-1β, thereby inhibiting NLRP3 inflammasome [55]; 3,4-DAA and Butyrate have also been found to affect the formation of NLRP3 inflammasomes through different pathways [56, 57].

## The role of NLRP3 inflammasome in diabetes

Although different types of diabetes have different etiologies, islet β cells damage is a common feature [58]. Recent studies have reported that the NLRP3 inflammasome is the critical substance in β cells dysfunction and death in type 1 and type 2 diabetes [59, 60]. NLRP3 can be activated by various substances such as glucose, uric acid, and cholesterol crystals and subsequently produce caspase-1, IL-18, and IL-1β, which promote islet inflammation, damage islet β cells, and reduce insulin secretion [61].

### TXNIP/NLRP3/IL-1β signaling pathway mediates β cell apoptosis

It is well known that the dysfunction of pancreatic β cells in T2D patients is closely related to the increase of β cells apoptosis. It is a relatively recognized mechanism that the thioredoxin-interacting protein (TXNIP)/NLRP3/IL-1β signaling pathway mediates the apoptosis of β cells for NLRP3 inflammasome to induce T2DM diabetes. TXNIP is an important factor expressed in β cells and regulates β cells apoptosis, inflammation, and oxidative stress. β cells themselves are capable of producing IL-1β independently of any viral infection or immune-mediated process, and elevated glucose concentrations induce islet beta cells to secrete more IL-1β. IL-1β triggers apoptosis by reducing I-κB expression and activating the transcription factor NF-κB, inhibiting β cell function and promoting Fas receptor upregulation. In addition to acting as a mediator of glucose-induced β cell apoptosis, IL-1β may also be involved in controlling pancreatic insulin reserve [62]. In type 2 diabetes, when β cells fail to compensate, it decreases insulin signaling and insulin-dependent glucose uptake, resulting in persistent hyperglycemia. After that, islets trigger the induction of ROS through the NADPH oxidase system, glucose induces the expression of TXNIP, and ROS triggers the dissociation of TXNIP from thioredoxin (TXN), thereby triggering the TXNIP-dependent activation of the NLRP3 inflammasome, and finally leads to the secretion of mature IL1β. Elevated IL-1β is an important cause of β cell death and dysfunction and decreased insulin secretion, contributing to decreased insulin secretion and aggravating hyperglycemia [63].

Recent research showed that another NLRP3-related cytokine, IL-18, was also positively associated with insulin resistance and increased risk of T2DM. However, the role of IL-18 in T1D is controversial. Some studies have shown that IL-18 may promote T1D development by inducing diabetic T-cell expansion in mice [64]. Other studies have proved that T1D development does not require IL-18 [65].

### Other NLRP3-related causes mediating β-cell apoptosis

In addition to TXNIP, the activation of NLRP3 inflammasome due to the increase of reactive oxygen species (ROS) production caused by other reasons is also one of the causes of diabetes. For example, in diabetic conditions, pancreatic β cell autophagic flux is blocked, resulting in intracellular accumulation of impaired organelles, generating a large amount of ROS, which in turn activates the NLRP3 inflammasome [66]. Donath found that advanced glycation end-products (AGEs) upregulate the protein expression level of the AGE pattern recognition receptor (RAGE), which can also increase ROS production, stimulate the activation of NLRP3 inflammasome, and lead to the activation of IL-1β. Subsequently, IL-1β secreted in the microenvironment exacerbates the chronic inflammatory response of islets [67]. Interestingly, administration of AGEs is also thought to significantly increase superoxide anion levels through upregulation of NADPH oxidase 2 (NOX2) protein, which generates increased expression levels of TXNIP and NLRP3 inflammasome components and protein interactions between TXNIP and NLRP3 [68], so we speculate that the two pathways leading to diabetes may be carried out together.

We found that there are a number of clinical factors affecting NLRP3 that have led to the study of diabetes. Youm’s study showed that pancreatic β cells in obese NLRP3-deficient mice were protected from inflammatory damage induced by a high-fat diet and were able to compensate by increasing insulin levels when blood sugar levels were higher than normal. At the same time, mtROS increases during obesity, and reactive oxygen species participate in the assembly of NLRP3 inflammasome. Mitochondrial dysfunction may affect the activation of NLRP3 inflammasome, leading to pancreatic injury in obese patients. Elimination of the NLRP3 inflammasome protects pancreatic β cells from cell death caused by prolonged high-fat feeding during obesity, and activation of the NLRP3 inflammasome in diet-induced obesity is a key trigger for pancreatic damage and an important mechanism for the progression of type 2 diabetes [69]. In addition, plasma IL-18 induced by NLRP3 was also positively associated with insulin resistance and an increased risk of type 2 diabetes [70]. Some substances, such as IAPP [71] and crystalline substances [72], were identified as possible triggers of inflammasome activation in type 2 diabetes.

### Other points of view

Although most experiments have shown that NLRP3 plays an important role in the development of diabetes, some believe that the absence of NLRP3 does not affect blood glucose levels or the apoptotis of β cells, suggesting that the NLRP3 inflammasome does not contribute to β cell function loss [73]. Experiments by Gurzov showed that mouse islets incubated with IL-1β alone did not exhibit β cell apoptosis [74]. Therefore, whether NLRP3 comes into play in the course of diabetes needs to be further studied in the future.

### The role of NLRP3 inflammasome in the treatment of diabetes

In treating diabetes, some substances related to NLRP3 inflammasome can inhibit the occurrence and development of diabetes in different ways (Table 2). We divide them into four categories.

**Table 2:** Drugs that affect the development of diabetes by interfering with the NLRP3 inflammasome and its associated molecules.

DRUG	TYPE	THERAPY PATHWAY	PMID
Arglabin	Natural compound	Inhibit NLRP3 by autophagy	27044804
Urolithin A	Natural metabolite	Activate autophagy to inhibit TXNIP/NLRP3 IL-1β inflammatory signal	34656886
Ginsenoside Rg1	Traditional Chinese medicine	Reduce inflammatory factors IL-1β and IL-18, and weaken the function of NLRP3	31738935
MCC950	Sulfonylureas	Inhibit the activation of NLRP3 inflammasome and Ang-II, decrease of IL-1β, and decrease islet β cell apoptosis	30939192
Glyburide	Sulfonylureas	Inhibit the activation of NLRP3 inflammasome	28703484
Astragaloside-IV (AS-IV)	Traditional Chinese medicine	Inhibit the activation of NLRP3 inflammasome	31558153
Grape seed procyanidinB2 (GSPB2)	Natural plant extract	Regulating IL-1β and NLRP3	26207855
Wu-Min-Wan	Traditional Chinese medicine	Decrease the expression levels of NLRP3, the adapter protein ASC, and caspase-1	30704457
Oridonin (Ori)	Natural plant extract	Block the interaction between NLRP3 and NEK7, inhibit the activation of NLRP3 inflammasome	33388733

#### TXNIP Inhibitors

TXNIP inhibitors are effective therapeutic agents for diabetes. Urolithin A, as a natural metabolite, significantly inhibited TXNIP expression, NLRP3 inflammasome activation, and IL-1β levels in β cells. Moreover, it inhibited glycolipid toxicity through the AMPK pathway and autophagy activation [63].

#### Inflammasome activation inhibitors

Arglabin, a natural product isolated from Artemisia glabella, controls inflammasome activation by inducing autophagy and inhibits the conversion of pro-IL-1β to bioactive IL-1β in a concentration-dependent manner in T2DM development Maturation pathway [70]. Astragalus (AS-IV) can suppress NLRP3 inflammasome activation in gestational diabetes pancreas by inhibiting the NF-κB pathway [75]. Wu-Mei-Wan, reduces the protein expression in NLRP3 as well reduces that of other inflammasome components, such as ASC and caspase-1 [76]. Oridonin (Ori) directly interacts with NLRP3 molecular binding, antagonism inhibits the function of the interaction between NLRP3 and NEK7, affects the activation of NLRP3 inflammasome [77]. In addition, the NLRP3 inflammasome inhibitor MCC950 is deemed to inhibit not only inflammasome activation but also Ang II-induced IL-1β elevation and apoptosis [78].

#### Inflammatory factor inhibitors

Some drugs improve the level of diabetes by lowering the concentration of inflammatory factors: Ginsenoside Rg1 was found to reduce the level of inflammatory factors IL-1β and IL-18 in the mouse T1DM model, promote insulin secretion, and weaken the function of NLRP3 in mouse liver and pancreas, is a potential drug for preventing the development and progression of T1DM [79].

#### Glyburide

Glyburide, also known as glibenclamide, is an NLRP3 inhibitor of sulfonylurea drugs, widely used in treating type 2 diabetes. It blocks potassium K^+^ channels on the membrane of pancreatic beta cells, preventing the efflux of K^+^ from cells [80]. At present, this drug has been widely used in the United States to treat T2D [81].

## Role of the NLRP3 inflammasome in chronic pancreatitis and pancreatic ductal adenocarcinoma

In addition to acute pancreatitis and diabetes, NLRP3 inflammasome has also become the object of attention in the research of chronic pancreatitis (CP) and pancreatic cancer recently.

### The role of NLRP3 in chronic pancreatitis

Similar to acute pancreatitis, Kanak demonstrated for the first time the role of the inflammasome in the pathogenesis of CP. Typical DAMPs such as HMGB-1 or ATP released by damaged acinar cells might lead to the assembly and activation of NLRP3 inflammasome, thereby exacerbating inflammation during CP. At the same time, it was proved that when the NF-κB pathway is suppressed, the inflammasome expression will be reduced, thereby reducing the severity of chronic pancreatitis [82].

Several drug studies targeting NLRP3 inflammasome therapy have been conducted in animal trials (Table 3). Similar to acute pancreatitis, Withaferin A (WA) acts as an inhibitor of NF-κB, blocking ER stress and NLRP3 inflammasome [82]. Total flavonoids from Psidium guajava leaves (TFPGL) significantly reduce inflammatory cell invasion and fibrosis. The expression of NLRP3 and caspase-1 was significantly decreased at both gene and protein levels. The expression of IL-1β and IL-18 was decreased [83]. P2X7R antagonist, Xiao Chai Hu Tang (XCHT) and Morus alba root bark (MEMARB) also inhibit chronic pancreatitis by decreasing the expression of NLRP3 in pancreas [84–86].

**Table 3:** Drugs that affect the development of chronic pancreatitis by interfering with the NLRP3 inflammasome and its associated molecules.

DRUG	TYPE	THERAPY PATHWAY	PMID
Withferin A	Natural plant extract	Block ER stress and NLRP3 inflammasome	27418337
Total flavonoids from Psidium guajava leaves (TFPGL)	Traditional medicinal plant	Decreasing the expression of NLRP3 and caspase-1	34961420
Oxidized ATP (OxATP)	P2X7R antagonist	Decreasing the expression of NLRP3 and caspase-1	28930866
Brilliant blue G (BBG)	P2X7R antagonist	Decreasing the expression of NLRP3 and caspase-1	28930866
Xiao Chai Hu Tang (XCHT)	Traditional Chinese medicine	Decreasing the expression of NLRP3	36096349
Morus alba root bark (MEMARB)	Traditional Asian medicine	Decreasing the expression of HSP70 and NLRP3-ASC	30335608

### The role of NLRP3 in pancreatic ductal adenocarcinoma

The role of NLRP3 in various cancers has been widely studied. Kantono found that the inflammasome is responsible for the complex biological mechanism of mediating inflammation in cancer cells [87]. NLRP3 is highly expressed in pancreatic ductal adenocarcinoma (PDA) cells and tissues, and knockdown of NLRP3 can reduce the proliferation, invasion and EMT (epithelial–mesenchymal transition) of cancer cells in vitro [88]. PDA may be related to NLRP3 polymorphism [89]. PDA is caused by chronic inflammation and driven by persistent inflammation associated with immunosuppressive CD4^+^ T cells. NLRP3 signaling drives CD4^+^ T cell differentiation into tumor-promoting type 2 helper T cells (Th2 cells), Th17 cells, and regulatory T cell populations, while inhibiting Th1 cell polarization and cytotoxic CD8^+^ T cell activation. Hu’s study showed that down-regulating NLRP3 inhibits PDA progression and reduces cell invasion induced by epithelial mesenchymal transformation [88]. An immunohistochemical and survival analyses showing that high NLRP3 expression was associated with lower survival and poorer prognosis in these patients, possibly due to an ineffective immune system response and increased tumor-promoted inflammation [90]. Research shows that inhibition of NLRP3 inflammasome activation by the specific NLRP3 antagonist MCC950 reduces cell viability in pancreatic cancer cells; however, the efficacy of MCC950 varies by cell type [91].

Das has confirmed that the TLR4/NLRP3/IL-1β inflammasome signaling axis might be the pathway through which it affects early pancreatic tumors. Tumor-derived IL-1β established an immunosuppressive cell population mediated by M2 macrophages, myeloid-derived suppressor cells, CD1d^hi^CD5^+^ regulatory B cells, and Th17 cells. When the absence of tumor cell-derived IL-1β signal in the tumor stroma enables CD8^+^ cytotoxic T cells to infiltrate and activate in the tumor, reducing the growth of pancreatic tumors, playing a significant part in the development and invasiveness of pancreatic ductal adenocarcinoma [88, 92]. IL-1β production by PDA-associated myeloid cells may also support tumor progression by promoting immune tolerance [92]. IL-18 can enhance the Th1 type immune response, thus producing a large amount of INF-γ and TNF-α, promoting chronic inflammation and forming the basis of tumor occurrence [93, 94]. Carbone conducted a study on 58 patients with pancreatic cancer and found that the expression of IL-18 in tumor tissues and serum was up-regulated, and the increase of serum IL-18 was associated with poor prognosis [95].

## Conclusion

As important diseases in the digestive system, pancreas-related diseases have brought many troubles to human beings in treatment. The gradual in-depth research on the NLRP3 inflammasome has made the corresponding pathway and the molecules on the pathway therapeutic targets for pancreatic diseases. This work comprehensively reviews the research status, mechanism of action, and drugs used in related treatments of NLRP3 inflammasome in four specific pancreas-related diseases, hoping to provide a logical and comprehensive summary article for relevant personnel.

Through summarizing the existing literature, we found that although the role of NLRP3 inflammasome in acute pancreatitis and diabetes is still controversial, the general mechanism is precise. We found that NLRP3 studies in pancreatic cancer and chronic pancreatitis are still at the basic stage. This may be the hotspot of future research in this field. NLRP3 may also be a key factor in the transformation of inflammation and cancer in the pancreas.
